# CAR-T cells and CAR-Treg cells for treatment of inflammatory bowel diseases

**DOI:** 10.1016/j.ero.2026.100224

**Published:** 2026-08-17

**Authors:** Markus F Neurath

**Affiliations:** Department of Medicine 1 & Deutsches Zentrum Immuntherapie DZI, Friedrich-Alexander-University Erlangen-Nürnberg, Erlangen, Germany

## Abstract

Inflammatory bowel diseases (IBD), including Crohn’s disease (CD) and ulcerative colitis (UC), are therapeutically challenging chronic inflammatory diseases of the intestine. Despite major advances in biologic and small-molecule therapies, many patients with IBD experience primary nonresponse, secondary loss of response, or severe complications, underscoring the need for innovative therapeutic strategies that lead to sustained immune recalibration. Chimeric antigen receptor (CAR)-based cellular therapies represent an emerging new concept for transformative medicine. These CAR-T cells are engineered to recognise defined surface antigens, such as CD19, and are therefore ideally suited to eliminate selected pathogenic immune cell populations, such as B lymphocytes. In IBD, this concept has gained momentum through evidence implicating mucosal B cells, plasmablasts, and disease-associated humoral responses as potential disease drivers in UC. A recent case report suggested that CD19-directed CAR-T cell therapy may induce profound remission in multirefractory UC. In parallel, CAR regulatory T cells (CAR Tregs) may offer a complementary approach for IBD therapy by redirecting suppressive immune function in the inflamed intestine. Particularly, interleukin-23 receptor-targeted CAR Tregs provide a mechanistically attractive strategy to modulate T helper–type 17-driven mucosal inflammation in CD. Safety considerations and translational challenges are potential concerns with regard to CAR-T and CAR-Treg therapies in IBD. However, these technologies may enable precision immune engineering for selected refractory disease endotypes in IBD.

## INFLAMMATORY BOWEL DISEASES

Inflammatory bowel diseases (IBD), comprising Crohn’s disease (CD) and ulcerative colitis (UC), are chronic immune-mediated disorders in which sustained tissue inflammation arises in genetically susceptible hosts. Host genetics, intestinal dysbiosis, altered epithelial barrier function, and proinflammatory mucosal immunity (e.g. via T helper 17 cytokine responses) play a key role in IBD pathogenesis [1,2]. The therapeutic armamentarium has expanded substantially during the past 2 decades, with neutralising monoclonal antibodies and small molecules targeting tumour necrosis factor (TNF), interleukin (IL)-12/23 p40, IL-23 p19, alpha4/beta7-integrins, sphingosine-1-phosphate receptor signalling and Janus kinase 1/3 pathways [[3], [4], [5], [6], [7], [8]]. Nevertheless, a considerable proportion of patients either fail to respond initially (primary nonresponse), lose response over time (secondary nonresponse), or develop severe complications despite advanced therapies [9,10]. In this context, cellular immunotherapies are increasingly viewed as a conceptual shift from selective pathway inhibition towards targeted immune system reprogramming [11]. Among the most innovative approaches are chimeric antigen receptor (CAR) T cells and CAR-engineered regulatory T cells (CAR Tregs), which offer the possibility of either eliminating selected pathogenic immune compartments or restoring immune tolerance within inflamed tissue [[12], [13], [14], [15], [16]]. Although these techniques have been initially tested in patients with cancer, it has recently been recognised that they also have great potential for the treatment of patients with autoimmune and chronic inflammatory diseases such as systemic lupus erythematosus.

## CAR-T CELLS: STRUCTURE AND FUNCTION

CAR-T cells are specific T lymphocytes that have been genetically modified to express a synthetic receptor with predefined antigen specificity (eg, CD19). A typical receptor contains an extracellular antigen-binding domain, usually derived from an antibody fragment (single-chain variable fragment), linked through hinge and transmembrane regions to intracellular signalling domains (CD3zeta chain, costimulatory domains from 4-1BB or CD28) that activate the T cell upon antigen engagement [16,17]. This design allows CAR-T cells to recognise surface molecules independently of classical antigen presentation by major histocompatibility complex molecules. After collection from the patient (autologous cells) or from an allogeneic donor (allogeneic cells), T cells are engineered (usually by lentiviral infection), expanded under controlled good manufacturing practice (GMP) conditions, and reinfused into the patients. Once they encounter cells expressing the predefined target antigen, CAR-T cells become activated and can induce cytotoxicity, proliferate, and persist over several months [[18], [19], [20]]. Hereby, CAR-T cells selectively eliminate predefined target cell populations in the host.

Initial clinical studies using CD19-directed CAR-T cell therapy were conducted in B cell malignancies (eg, leukaemia, lymphomas) [17]. This remarkable clinical success has led to increasing interest in autoimmune disease, where pathogenic B cell and plasmablast populations can sustain chronic immune activation [15,16]. Recent data in systemic lupus erythematosus and other antibody-mediated autoimmune diseases suggest that CD19 CAR-T cells can produce profound B-lineage depletion and durable treatment-free remission in selected patients [16,21]. In this context, CAR-T cells induce highly effective depletion of B cells in inflamed tissues, leading to an immune reset characterised by markedly reduced or absent inflammation. In some patients with systemic lupus erythematosus, a single CAR-T cell treatment has led to drug-free clinical remission over several years and potentially even cure of the disease [16], [21]. This observation could be relevant to IBD because it raises the possibility that selected intestinal inflammatory phenotypes may be maintained by similarly targetable immune cell compartments.

## CAR-T CELLS AS A POTENTIALLY NEW TREATMENT OPTION IN IBD

**Figure 1:**
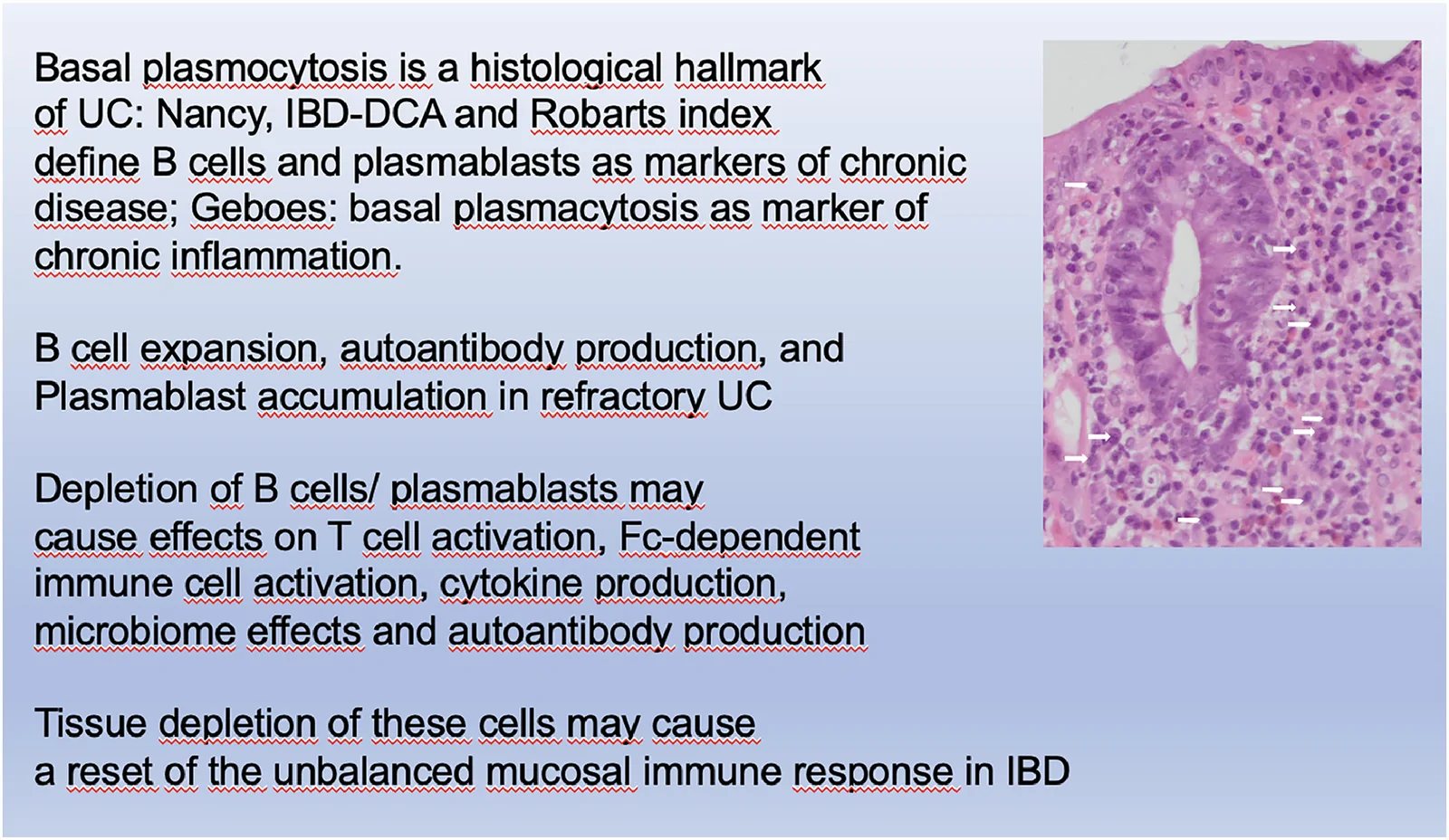
Evidence for the role of B cells and histopathological scoring systems for ulcerative colitis (UC) and detection of plasma-cell–rich chronic inflammation. Representative histologic image of colonic mucosa showing chronic inflammatory infiltrates, including plasma cells (right side, arrows). Left side: established histopathologic scoring systems for UC, including the Nancy Histological Index, Robarts Histopathology Index, IBD-DCA (Distribution/Chronicity/Activity) score, and Geboes score, incorporate chronic inflammatory infiltrates and/or basal plasmacytosis as markers of chronic mucosal inflammation. These observations underscore the relevance of plasma-cell infiltration as a key histologic component in the grading of UC. IBD, inflammatory bowel disease.

The rationale for CAR-T cells in IBD depends on the identification of disease-driving cell populations that can be selectively targeted. Various studies on IBD pathogenesis have highlighted T cell activation, fibroblast and innate immune dysregulation, epithelial barrier failure, and microbial imbalance as key drivers of chronic intestinal inflammation [[22], [23], [24], [25]]. In contrast, B cells have been largely neglected as drivers of disease activity, as clinical trials targeting B cells using anti-CD20 antibodies (rituximab) showed disappointing results. However, accumulating evidence indicates that B cells and antibody-producing cells may contribute to disease chronicity, particularly in UC [10]. Basal plasmacytosis has been identified as a characteristic histologic feature of chronic UC, and inflamed colonic tissue may contain expanded populations of activated CD19-positive plasmablasts and plasma-cell precursors (Fig 1) [10]. Therefore, the presence of plasma cells is an important component of many histologic scores for colitis activity in UC. In addition, UC has been associated with autoantibodies directed against epithelial or adhesion-related antigens, including antibodies against αvβ6 integrin, which have been linked to disease activity and aggressive disease behaviour in recent studies [[26], [27], [28]].

Histologic scores underscore the potential relevance of plasmablasts and plasma cells in UC. In fact, the histologic assessment of UC is based on established scoring systems that capture both inflammatory activity and chronicity. In addition to neutrophilic activity, cryptitis, crypt abscesses, and erosions, the chronic inflammatory infiltrate is an important component of evaluation. Plasma cells, and particularly basal plasmacytosis, are regarded as key histologic features of chronic mucosal inflammation (Fig 1). In the Nancy Histological Index, chronic inflammation, including plasmacytosis, is incorporated into the grading of the chronic inflammatory infiltrate [29,30]. Moreover, the Robarts Histopathology Index likewise includes plasma-cell–rich chronic inflammatory infiltrates in its histologic assessment [29,31]. Furthermore, the IBD-DCA (Distribution, Chronicity, Activity) score gives special emphasis to basal plasmacytosis by evaluating it independently in the context of chronicity [32]. Finally, the Geboes score also incorporates plasma cells within the chronic inflammatory infiltrate. Thus, plasma-cell accumulation is not an isolated finding but a recurring element across several validated histologic scoring systems for UC [29]. Its assessment contributes to the evaluation of chronicity, disease duration, and persistent mucosal immune activation.

Early clinical trials targeting B cell function used the anti-CD20 antibody rituximab in UC. The negative outcome of these studies questioned the clinical relevance of B cell targeting in IBD. However, the failure of conventional B cell depletion with anti-CD20 antibodies does not necessarily invalidate B cells as therapeutic targets in IBD [33,34]. Rituximab primarily removes CD20-positive B cells but has limited activity against plasmablasts and differentiated plasma cells, and it may not adequately deplete disease-relevant mucosal B-lineage cells [35]. Thus, rituximab may efficiently deplete peripheral B cells but not mucosal B cells in the inflamed intestine. In contrast, CAR-T cells may achieve a more complete depletion of CD19-expressing mucosal cells, including activated effector memory B cells and mucosal plasmablasts. This difference is potentially relevant, as tissue-resident or inflammation-associated B-lineage networks in the mucosa can sustain chronic intestinal inflammation.

A recent report of CD19-directed CAR-T cell therapy in a patient with severe multidrug-resistant UC underscored the potential relevance of mucosal B cell depletion in UC [36]. In this case with multirefractory disease, deep mucosal CD19-directed targeting was associated with drug-free clinical remission, endoscopic remission, histologic healing, and markedly suppressed disease-associated humoral activity. Importantly, integrin αvβ6 autoantibody levels were suppressed after CAR-T cell therapy, highlighting a profound effect of CAR-T cells on mucosal B cell and plasmablast activity [36]. Although a single case cannot establish efficacy of CAR-T cell therapy in UC, this case report provides the first proof of concept that B-cell-directed cellular immunotherapy can coincide with resolution of otherwise refractory intestinal inflammation. It also suggests that CAR-T cell therapy could define specific roles of individual immune cell subsets in UC *in vivo*.

## POTENTIAL CAR-T CELL TARGETS IN IBD BEYOND CD19

CD19 is only 1 possible entry point for CAR-T cell therapy. In particular, it has been recently shown that BCMA (B cell maturation antigen, TNFRSF17)-directed CAR-T cells may target more differentiated antibody-producing cells, including plasma-cell compartments, and could be relevant in clinical situations in which chronic inflammation is dominated by pathogenic antibody production [37]. This is due to the fact that BCMA is highly expressed on plasma cells and long-lived plasma cells, whereas CD19 expression is low in plasma cells and very low or absent in long-lived plasma cells. However, such a BCMA-directed approach may carry increased risk of hypogammaglobulinaemia and impaired mucosal antibody defence. Therefore, the chances and risks of such treatment for IBD must be carefully considered. CD7-directed CAR-T cells, currently being explored in some inflammatory and autoimmune settings, could also theoretically eliminate activated T cell subsets in IBD [38]. However, broad T cell depletion in IBD is potentially hazardous because intestinal barrier integrity and gut homeostasis depend on tightly regulated protective immunity. Furthermore, stromal targets such as fibroblast activation protein may become relevant in fibrostenotic CD, where activated fibroblasts contribute to extracellular matrix deposition and irreversible tissue remodelling. In this setting, targeting tissue repair programmes in the intestine will require particular caution to avoid impairing wound healing [[39], [40], [41]].

## SAFETY CONSIDERATIONS FOR CAR-T CELLS IN IBD

The use of cytotoxic CAR-T cells in chronic inflammatory intestinal disease raises substantial safety questions. Conventional CAR-T cell protocols often require lymphodepleting chemotherapy to facilitate expansion and persistence of transferred T cells [15]. In IBD, such preconditioning may increase infectious risk, particularly in patients with compromised epithelial barrier function, bacterial translocation, malnutrition or prior immunosuppression. Acute toxicities such as cytokine release syndrome (CRS) and immune effector cell-associated neurotoxicity syndrome (ICANS) must also be considered, even if autoimmune indications may have markedly lower pathogenic cell burden than haematologic malignancies [15,16]. Furthermore, it should be considered that long-term B cell depletion could impair vaccine responses, reduce protective immunoglobulin levels, and alter secretory immunoglobulin A–mediated host–microbiota homeostasis. These issues are especially relevant in the gut, where immune protection and immune tolerance are deeply interdependent.

## CAR TREGS FOR IBD THERAPY

CAR Tregs represent a biologically distinct form of immune engineering using CAR cells. Instead of redirecting cytotoxic T cells to eliminate target cells, CAR Tregs are regulatory T cells (Treg cells) modified to recognise selected antigens and deliver suppressive activity at defined tissue sites [42,43]. Conventional Treg cells maintain immune homeostasis through various mechanisms, including IL-10 and TGF-β production, inhibition of antigen-presenting cells, metabolic disruption of effector T cell activation, and stabilisation of tolerance at barrier surfaces [44,45]. In IBD, endogenous Tregs are often present in inflamed tissue but appear insufficient to counteract the magnitude, persistence, or inflammatory resistance of effector responses [23,46,47].

CAR engineering could improve Treg therapy by providing antigen-directed activation and tissue localisation [42,43]. In principle, CAR Tregs might accumulate within inflamed intestinal niches, become activated by disease-relevant antigens, and suppress local immune pathology without inducing broad systemic immunosuppression. This makes them particularly attractive for IBD, where complete immune ablation is rarely desirable and where localised restoration of tolerance may be more appropriate than elimination of entire immune cell lineages.

One of the most compelling IBD-relevant CAR-Treg strategies targets the IL-23 receptor. IL-23 signalling is central to pathogenic T helper–type 17 (Th17) immunity and contributes to the activation of Th17 cells, Tc17 cells, γδ T cells, and group 3 innate lymphoid cells [42]. IL23R-specific CAR Tregs have been designed to recognise IL-23R-expressing inflammatory cells and exert regulatory functions in their vicinity. Preclinical studies indicate that such cells can retain regulatory identity during expansion and respond to IL-23R-positive target environments [42]. This approach is conceptually elegant because it converts a validated inflammatory pathway into a spatial cue for tolerance induction. Rather than systemically blocking IL-23, IL23R-CAR Tregs may concentrate suppressive activity where IL-23-dependent immune activation is most pronounced. The clinical validation of this approach is therefore of considerable interest.

## TRANSLATIONAL HURDLES FOR CAR-TREG CELLS

Several barriers must be overcome before CAR Tregs can enter routine IBD treatment [42,43,48]. First, engineered Tregs must preserve lineage stability in inflamed tissue rich in IL-6, IL-1β, TNF, and IL-23, because loss of FOXP3 stability or acquisition of effector-like properties would represent a major safety concern. Second, they must reach and persist in the intestinal mucosa, which may require additional engineering of gut-homing receptors or tissue-retention programmes. Third, manufacturing remains demanding, as Tregs are less abundant than conventional T cells, expansion protocols must be highly controlled, and contaminating effector T cells must be rigorously excluded. Finally, the choice of target antigen remains critical. Ideal targets should identify inflammatory tissue or pathogenic cell populations without triggering systemic immune suppression or off-target effects.

## FUTURE POTENTIAL OF CAR-T CELL APPROACHES IN IBD

The future role of CAR-based therapies in IBD may be limited to selected patient populations [21,49]. Currently, these technologies are unlikely to replace biologics for most patients with early or moderate disease, given that CAR-based therapies are costly, technically complex, and their safety and efficacy profiles remain insufficiently characterised [5,[50], [51], [52], [53]]. Their greatest potential lies in endotype-defined, treatment-refractory settings in which conventional pathway blockade is insufficient. CD19-directed CAR-T cells may be most relevant for IBD phenotypes with prominent mucosal plasmablast expansion, lymphoid aggregates, or disease-associated autoantibody signatures. In contrast, CAR Tregs may be better suited for IBD phenotypes driven by persistent T helper–type 17 immunity, tissue-localised inflammation or failure of mucosal tolerance. Stromal or fibrosis-directed CAR approaches could eventually address fibrostenotic disease, an area of profound unmet need [48].

The field is also moving rapidly towards safer and more scalable platforms [37,49,54,55]. Emerging approaches include transient RNA-based CAR expression, allogeneic products, in vivo CAR engineering using targeted lipid nanoparticles, and strategies that avoid lymphodepletion, potentially improving scalability and safety [56]. For IBD, these developments are especially important because the acceptable risk threshold is lower than in oncology. Future trials should integrate deep mucosal phenotyping, spatial biology, immune repertoire analysis, microbial profiling, and long-term safety monitoring. Clinical endpoints should extend beyond symptom control to include endoscopic healing, histologic remission, steroid-free and drug-free remission, preservation of barrier immunity, and evidence of immune reprogramming.

In summary, CAR-T cells and CAR Tregs represent 2 complementary new therapeutic concepts for IBD [57]. Although CAR-T cells may enable precise deletion of pathogenic immune compartments, CAR Tregs may restore localised immune regulation. Both approaches remain early in development for IBD, but they have the potential to redefine treatment for selected patients with otherwise refractory disease. Controlled clinical trials are needed to unequivocally define the safety and efficacy of these approaches for IBD therapy.

## Funding

MFN has been supported by grants from the DFG/Deutsche Forschungsgemeinschaft (TRR241, TRR417, SFFB1181), the Rainin Foundation Innovator Award, and IZKF/Interdisziplinäres Zentrum für Klinische Forschung, Erlangen.

## CRediT authorship contribution statement

**Markus F Neurath:** Conceptualization, Writing – original draft, Writing – review & editing.

## Competing interests

MFN is advisor to Abbvie, Boehringer, J&J, Pentax, Cabaletta, Tr1x, Takeda and Pfizer.
